# Optimal Use of Drain Tubes for DIEP Flap Breast Reconstruction: Comprehensive Review

**DOI:** 10.3390/jcm13216586

**Published:** 2024-11-01

**Authors:** Bryan Lim, Ishith Seth, Konrad Joseph, Jevan Cevik, Henry Li, Yi Xie, Axel Mendoza Hernandez, Roberto Cuomo, Warren M. Rozen

**Affiliations:** 1 Department of Plastic Surgery, Peninsula Health, Melbourne, VIC 3199, Australia; ishith.seth@monash.edu.au (I.S.); jevan.cevik@monash.edu (J.C.); henry.li@easternhealth.org.au (H.L.); yxie@phcn.vic.gov.au (Y.X.); warrenrozen@psvic.com.au (W.M.R.); 2 Department of Surgery, Port Macquarie Base Hospital, Port Macquarie, NSW 2444, Australia; 3 Department of Medicine and Plastic Surgery, Universidad del Valle de Mexico, Zapopan 45010, Mexico; axelmanuel.mendoza@my.uvm.edu.mx; 4 Department of Medicine, Plastic Surgery and Neuroscience, University of Siena, 53100 Siena, Italy; roberto.cuomo2@unisi.it

**Keywords:** DIEP, breast reconstruction, drain tubes, closed suction drain, timing

## Abstract

**Background**: Deep inferior epigastric perforator (DIEP) flap breast reconstruction is an increasingly popular technique, but controversy exists regarding the optimal use of closed suction drains (CSD) at the abdominal donor site. This narrative review synthesizes current evidence on CSD application, criteria for placement/removal, and complications in DIEP flap procedures. Alternative techniques and implications for postoperative care are also discussed. **Methods**: A systematic search was conducted in August 2024 across several databases to identify English language studies related to CSD use in DIEP flap breast reconstruction. Inclusion criteria consisted of original research on aspects such as CSD volume criteria, timing, complications, alternatives like progressive tension sutures, and impact on showering and patient outcomes. References from relevant papers were hand-searched. **Results**: The review found a lack of consensus on CSD protocols, with drainage volume triggering removal varying widely from 5 mL to 80 mL daily. While CSD may reduce seroma/hematoma formation, earlier removal (≤3 days) did not increase complications and shortened hospital stay. Progressive tension sutures show promise as an alternative, with evidence of comparable or reduced complications and improved recovery versus CSD. The safety of early showering with drains remains unclear. **Conclusions**: Although CSD aims to minimize postoperative complications, more rigorous randomized trials are needed to establish evidence-based practices for the timing of removal and demonstrate the efficacy of emerging drain-free techniques on patient-centered outcomes. Standardized criteria could reduce practice variability. Further research should also explore the long-term impact of drainage strategies on aesthetic and functional results.

## 1. Introduction

Autologous breast reconstruction utilizing the deep inferior epigastric perforator (DIEP) flap represents a significant advancement in post-mastectomy care, offering congruent aesthetic outcomes with reduced donor site morbidity [1,2,3]. In 2020, the United States saw 23,324 out of 137,808 breast reconstruction patients opting for the DIEP flap technique [4]. Despite its popularity, the postoperative management, specifically the application of closed suction drainage (CSD) at the donor site, remains contentious within the surgical community [4,5,6].

The deployment of CSD in DIEP flap breast reconstruction is conventionally justified by its potential to attenuate seroma formation and other complications by minimizing dead space and preventing fluid accumulation [3]. However, the practice is not without criticism; drain use may contribute to increased infection risks, pain, restricted mobility, and protracted hospital admissions, all of which have prompted the exploration of alternative, drain-free closure techniques [7,8]. Divergent surgical preferences further complicate the scenario, with drainage removal criteria varying widely, from daily volumes of 5 mL to 80 mL [6,9,10]. This heterogeneity underscores the lack of consensus and standardized protocols within the field [10]. Moreover, the potential benefits of early postoperative showering for patient comfort, juxtaposed against the uncharted safety of such practices in the context of CSD, warrant systematic investigation.

Therefore, this narrative review aims to distill and integrate the multifaceted literature on CSD application, focusing on the criteria for placement, duration, and removal in the context of DIEP flap breast reconstruction. Additionally, we aim to scrutinize the existing evidence for alternative techniques such as progressive tension sutures and barbed sutures, to assess their efficacy in reducing complications, contemplate their impact on patient outcomes and hospital resource allocation; and the implications of postoperative showering practices for patients with CSD, a topic scarcely addressed in the current literature.

## 2. Materials and Methods

PubMed, Web of Science, EMBASE, Scopus, and the Cochrane Library databases were searched from their inception until August 2024 by two independent authors to ensure thoroughness and reduce selection bias. The keywords and phrases utilized in the search were “DIEP flap”, “breast reconstruction”, “autologous breast reconstruction”, “closed suction drainage”, “CSD”, “drain”, “drainage volume”, “postoperative care”, “seroma”, “wound dehiscence”, “infection”, “postoperative showering”, and “patient outcomes”. The references of prior reviews and the studies included in our initial search were used to identify any additional pertinent literature.

Our inclusion criteria consisted of English language peer-reviewed original research articles, systematic reviews, meta-analyses, and clinical trials. The studies were required to specifically address the use and impact of CSD in DIEP flap breast reconstruction in human participants, encompassing aspects such as drainage volume criteria for removal, timing of drain removal, complications associated with CSD use, alternative techniques to CSD, and the influence of CSD on postoperative showering and patient outcomes. We omitted articles not published in English, non-peer-reviewed literature such as opinion pieces, editorials, and letters to the editor, studies not directly related to the use of CSD in DIEP flap breast reconstruction or its alternatives, and studies based on animal models.

A standardized narrative review checklist (Supplementary File S1) included the following:

- Clear definition of the research question.
- Systematic search strategy with defined databases and keywords.
- Inclusion and exclusion criteria to identify relevant studies.
- Evaluation of the quality and relevance of the studies.
- Synthesis of the findings to provide a coherent narrative.
- Discussion of the implications of the findings for clinical practice.
- Identification of gaps in the literature and suggestions for future research.

## 3. Results and Discussion

### 3.1. Rationale for Closed Suction Drains

Closed suction drains are commonly used during breast reconstructive procedures using DIEP flaps to help prevent the accumulation of fluid and reduce the risk of potential complications (Table 1).

Intraoperatively, particularly if DIEP reconstructive surgery immediately follows a mastectomy, native tissue at the recipient site, such as blood vessels and lymphatics, undergoes a degree of trauma which can lead to the accumulation of fluid (e.g., blood, serum, or lymph) postoperatively under the skin flaps [11,12]. This accumulation of fluid gives rise to collections of seromas or hematomas which can apply pressure to nearby structures. One significant concern is compression of the flap pedicle leading to compromised flap viability [13,14]. Furthermore, increased pressure subcutaneously places the native mastectomy skin under tension, increasing the risk of skin necrosis or wound dehiscence [15,16]. Moreover, the presence of static fluid promotes a favorable environment for bacterial growth, leading to superimposed infection of these fluid collections, reduced tissue oxygenation, and often necessitating a return to theater for drainage [17,18].

In this context, closed suction drains provide a route for excess fluid to be removed in a controlled fashion in the early postoperative period. The drains are placed under the skin and apply gentle, continuous suction using a vacuum-sealed reservoir or connector to facilitate drainage via a small, perforated tube. This is thought to help reduce tension and swelling under the flaps by evacuating accumulations of fluid from dead space [19]. Reduced tension and swelling can help improve the blood circulation in the tissue, potentially enhancing flap or native skin survival. Moreover, drains may also provide quantifiable data to surgeons on fluid drainage volumes over time, which gives an indication about the development of potential complications such as bleeding. If output abruptly increases or becomes sanguineous, it may indicate a developing hematoma. Ongoing high output signals the possibility of a lymphatic leak. These data facilitate early intervention and may help minimize flap loss or other complication rates. Thus, closed suction drains have numerous potential benefits providing a rationale for their use in DIEP flap breast reconstruction. Interestingly, Skorochod et al.’s analysis of 743 patients revealed that the use of a single drain tube was associated with higher rates of seroma formation compared to cases without drain tubes [20]. Despite the differing results of that singular study, the majority of evidence supports the use of closed suction drains as an effective method for reducing hematoma and seroma formation. By enabling controlled fluid evacuation, minimizing tissue tension, and providing quantitative data on fluid dynamics, drains may enhance postoperative outcomes in DIEP flap breast reconstruction. While recognizing their limitations and potential complications, their role in managing fluid collection reinforces their continued application in this context.

### 3.2. CSD Placement Location in DIEP Flap Reconstruction

When the decision to place drains has been made, the location of placement is an important consideration. Traditionally, CSDs are placed at both the abdominal donor site and the recipient sites, often up to two drains per site [20,21,22]. This can leave patients with up to six drains in situ in the setting of bilateral DIEP reconstructions resulting in discomfort and limiting postoperative mobility. More recently, research has shown that the use of progressive tension sutures, particularly using barbed sutures, in the closure of the donor site may alleviate the need for donor-site drain placement, with studies showing improved or comparable postoperative donor site complications when this technique is compared to the use of drains alone [5,8]. At the recipient site, drains are typically placed laterally and inferiorly in a dependent position to allow effective drainage of fluid when the patient is supine or sitting up in bed. Furthermore, placement of the drain in this location places it away from the flap pedicle which is often medial. Another consideration is the appearance of scars following drain placement. Drain scar appearance is a commonly cited concern for patients who undergo DIEP flap reconstruction [23]. Patients may express individual preference for the drain site and involving them in the planning of drain placement may help alleviate their anxiety [23].

**Table 1:** Summary of all included literature on CSD use in DIEP breast reconstruction.

Study	Topic	Study Design	Sample Size	Outcomes/Findings
Phillips et al. 2011 [1]	Antibiotic use and CSD practice	Retrospective analysis	130	CSD reduces infection but lacks standardization in volume and duration for removal.
Ogawa and Tahara 2022 [4]	Postoperative showering with CSD	Retrospective cohort study	70	Showering with CSD is safe but long-term results on wound healing need further study.
Liang et al. 2016 [5]	Barbed sutures to reduce drainage	Prospective cohort study	50	Barbed sutures significantly reduce fluid drainage and decrease seroma rates.
Thacoor et al. 2018 [7]	Drain-free abdominal site DIEP outcomes	Retrospective study	109	Drain-free abdominal site closure reduces hospital stays without increased complications.
Nagarkar et al. 2016 [8]	Barbed sutures in DIEP closure	Retrospective review	93	Barbed progressive tension sutures improve recovery and reduce seroma without drains.
Miranda et al. 2014 [9]	Abdominal drains for DIEP reconstruction	Retrospective analysis	80	Variable CSD protocols based on surgeon preferences, affect outcomes inconsistently.
Skorochod et al. 2024 [20]	Abdominal drains for abdominoplasties	Retrospective cohort study	743	Insertion of single drain associated with lower risk of infections and wound dehiscence but a greater risk of seroma formation
Nishioka et al. 2020 [23]	Drain site selection and patient preference	Prospective study	68	Patient participation in drain site selection improves comfort and reduces anxiety.
Fracol et al. 2023 [24]	ERAS in DIEP flap with 24-h discharge	Prospective study	45	Enhanced Recovery After Surgery (ERAS) protocol safely discharges DIEP patients within 24 h.
Evgeniou et al. 2023 [25]	Drain-free DIEP concept	Prospective cohort study	47	Drain-free DIEP concept shortens hospital stay and avoids complications.
Mohan et al. 2015 [26]	Impact of drain-free donor site on patient outcomes	Retrospective review	93	No increase in complications observed with drain-free DIEP closure; faster recovery.
Pollock et al. 2000 [27]	Progressive tension sutures to reduce complications	Prospective study	100	Progressive tension sutures reduce the risk of seroma, postoperative pain, and infection.
Chan et al. 2020 [28]	No-drain technique in abdominal closure	Retrospective study	17	No-drain abdominal closure reduces hospital stays and complications, faster recovery.
Wu et al. 2020 [29]	Quilting sutures to reduce seroma	Retrospective analysis	235	Quilting sutures reduce the rate of seroma formation in mastectomy patients.
Lee et al. 2016 [30]	Quilting sutures in breast reconstruction	Randomized trial	80	Quilting sutures result in fewer complications and better postoperative outcomes.
Bhagchandani et al. 2023 [31]	Mastectomy flap quilting vs. conventional sutures	Prospective cohort study	70	Mastectomy flap quilting leads to reduced seroma, shorter hospital stay, and quicker recovery.
Shin et al. 2012 [32]	Quilting with fibrin sealant for seroma prevention	Retrospective study	20	Combining quilting sutures with fibrin sealant results in fewer seroma formations.
Eliav et al. 2021 [33]	Meta-analysis on quilting and seroma	Meta-analysis	50	Quilting sutures reduce seroma and hospital stay duration compared to conventional techniques.
Stoyanov et al. 2017 [34]	Review of drainage in mastectomy	Mini-review	60	Electrocautery contributes to seroma formation and increases complications postoperatively.
Porter et al. 1998 [35]	Electrocautery and seroma formation	Randomized trial	90	Electrocautery leads to higher rates of seroma formation compared to scalpel techniques.
Hashemi et al. 2004 [36]	Seroma post-mastectomy factors	Prospective study	120	Body mass index and dissection methods increase the risk of seroma formation.
Scevola et al. 2002 [37]	Drains and seromas in TRAM flap breast reconstruction	Prospective study	80	TRAM flap drains reduce seroma but cause more patient discomfort and extended stays.

The optimal number of drains for DIEP flap reconstruction remains inconclusive based on the literature. While studies like those by Philips et al. and Miranda et al. highlight the variability in CSD protocols based on surgeon preference [1,9], others indicate that alternative techniques, such as barbed sutures and drain-free closures, may be equally effective or superior [5,8,25]. Research by Thacoor et al. and Nagarkar et al. shows that drain-free techniques can reduce hospital stays and complications without compromising patient safety [7,8]. Additionally, Skorochod et al. found that a single drain is linked to lower infection rates but higher seroma formation, complicating drain placement decisions [20]. Overall, despite traditional support for drains, emerging evidence for drain-free methods indicates a need for standardized guidelines, with further research required to determine the optimal approach for postoperative management.

### 3.3. CSD Placement Duration and Removal in DIEP Flap Reconstruction

The duration that a drain is left in place, and the timing of its removal are important considerations in the postoperative care of a patient who has received a DIEP flap. While no consensus exists, typically, CSDs are kept in place until the output falls below a certain volume in a given time frame (often 20–50 mL over 24 h for two consecutive days) [7,9]. This criterion is, in theory, supposed to ensure that the risk of hematoma or seroma formation is minimized before the drain is removed. The timing of this removal therefore greatly varies between patients and depends on numerous factors including patient factors, surgeon preference, and the extent of the operation. Early removal of the drain can increase the risk of seroma/hematoma while late removal leads to prolonged discomfort and increases the risk of infection. Yet, it has been shown that the early removal of drains (within 3 days of operation) has been associated with decreased lengths of hospital stay, without increasing complication rates [9]. Furthermore, the proposed Enhanced Recovery After Surgery (ERAS) protocols for DIEP aim for discharge within 24–48 h and have been shown to be safe and effective amongst DIEP patients [24]. Ultimately, the postoperative monitoring of drain outputs and educating patients, along with healthcare staff, on drain care and the recognition of drain-related complications is essential for optimizing outcomes. Although the timing of drain removal is influenced by various factors and lacks a standardized approach, the literature suggests that early removal, when appropriate, can reduce hospital stays without raising complication rates. ERAS protocols further support this practice, promoting safe and effective discharge timelines.

### 3.4. Potential Complications of CSD Placement in DIEP Flap Reconstruction

**Figure 1:**
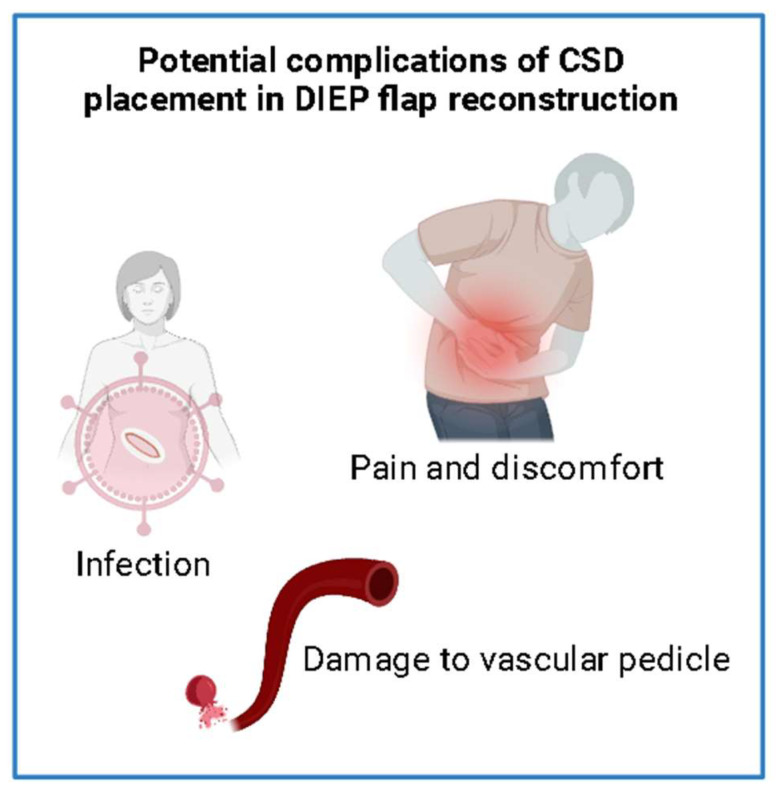
Potential complications of CSD placement in DIEP reconstruction.

The placement of a CSD for DIEP flap reconstruction can be associated with several potential complications (Figure 1). Infection, facilitated by the introduction of bacteria along the drain tract is a potential complication that can lead to significant morbidity including infected collections requiring debridement/washouts and possibly compromising the viability of the flap. Furthermore, another possible complication from the use of drains is the damage to the vascular pedicle itself. Conversely, Philips et al. reported reduced infection rates associated with closed suction drains (CSDs) in their sample of 130 patients, a finding corroborated by similar results from Skorochod et al.’s 743 patients [1,22]. Additional studies examining CSD use similarly indicate a decrease in overall complication rates [7,25], though further research has been recommended to enhance the reliability of these findings. Overall, the literature continues to support the use of closed suction drains (CSDs) as an effective measure in reducing infection and overall complication rates, underscoring their value in postoperative management.

Although rare, if the drain tube is placed too close to the vascular anastomosis or pedicle, inadvertent trauma can occur if it is inserted aggressively intraoperatively or if it moves postoperatively. Moreover, drains result in decreased mobility and increased patient discomfort which can lead to increased nursing requirements and extended hospital admissions increasing the risk of further complications whilst in hospital [7,8]. Careful patient selection and monitoring are essential to minimize risks and ensure optimal outcomes in patients receiving closed suction drains.

Research on the impact of CSDs on postoperative showering is limited, with only one relevant study identified [4]. Analyzing the results from 30 patients, Ogawa and Tahara found no significant differences in complication rates based on the number of CSD tubes connected to the abdominal donor site, indicating that postoperative showering does not increase risks [4]. Furthermore, the number of CSD tubes did not affect the timing of drain removal or when patients could begin showering. Interestingly, patients with two drains did not shower earlier than those with one or none, contradicting our initial hypothesis. Although a few complications were noted in patients with one remaining drain, statistical analysis revealed no significant differences among the groups, suggesting that patients with two or fewer drains can safely shower. Overall, their findings support the safety of early postoperative showering for patients with CSDs [4]. However, further studies are needed to thoroughly investigate the relationship between CSD use, timing of postoperative showers, and associated complications.

### 3.5. Alternative Options

Some studies suggest that drain-free abdominal closure in DIEP reconstruction can be achieved safely [7,8,25,26]. In 2018, Thacoor et al. found that donor-site drain-free DIEP reconstruction does not increase complication rates, and decreases inpatient hospital stays when compared with the same procedures involving drain placement (*n* = 109) [7]. In 2023, Evgeniou et al. tested a drainless DIEP flap concept across 47 patients and found that the drainless procedure led to shorter overall hospital stays without an increase in complication rates [25].

As previously mentioned, achieving abdominal closure with progressive tension sutures (PTSs) in abdominoplasty procedures has led to results comparable with CSD placement [27], and recent literature has confirmed the efficacy of PTS implementation in DIEP flap reconstructions [8,27,28]. In their 2015 retrospective review (*n* = 93), Mohan et al. found the use of PTSs for abdominal flap closure after DIEP flap harvest to be associated with decreased post-operative pain and earlier discharge without an increased risk of complications when compared with CSD [26]. In a 2020 retrospective study assessing 17 patients who underwent DIEP flap donor site closure with PTS, none developed a seroma and had faster discharge times than their counterparts who underwent conventional management with drains [26]. It should be recognized, however, that not all patients may be suitable for drain-free procedures. For example, patients with a body mass index above 30 kg/m^2^ may be at higher risk of complications in such scenarios [25]. The literature indicates that PTS can achieve outcomes similar to CSD in abdominal closures, but it is not definitive that PTS eliminates the need for drains in all patients. Individual factors, such as body mass index, may affect complication risks. Further research is needed to determine which patients can safely undergo drain-free PTS closure.

Quilting sutures are gaining popularity due to their ability to reduce the rates and volume of seromas [29,30,31,32,33]. Wu et al. retrospectively studied 235 breast cancer patients and found that the incidence of Grades 2 and 3 seromas were higher in the conventional suture group than in the quilt suture group (19.3% vs. 9.5%, *p* = 0.032) [29]. Bhagchandani et al. similarly discovered lower rates of seroma formation in their quilting suture group compared to the conventional suture group (23.26% vs. 57.58%, *p* = 0.002) and a shorter hospital stay (4.28 vs. 9.76 days, *p* = 0.0001) [31]. Further research has been suggested to investigate the amalgamation of quilting sutures with other preventative measures like fibrin sealants [33].

Electrocautery, on the other hand, offers contentious results. Stoyanov’s review found that harmonic scalpels produced less drainage and complications than electrocautery [34]. Additionally, randomized trials have shown that using electrocautery for dissecting flaps is highly associated with increased seroma formation compared to scalpel dissections post mastectomy [35,36]. Unfortunately, the current literature involving electrocautery and drain tube removal times remains scarce.

### 3.6. Limitations

This narrative review, while comprehensive in its current scope, encounters several limitations that warrant acknowledgment. Firstly, the majority of the included studies are retrospective in nature, which inherently limits the ability to establish causality and control for confounding variables. The retrospective design also often leads to a lack of standardized protocols across studies, particularly in terms of criteria for drain placement and removal, which complicates direct comparisons. Additionally, there is a notable paucity of RCTs within the literature, which are the gold standard for evaluating clinical interventions, thus a systematic review and meta-analysis could not be performed. The absence of such trials limits the strength of the evidence regarding the efficacy and safety of CSD versus drain-free techniques in DIEP flap breast reconstruction. Another limitation is the potential bias introduced by the variation in surgical techniques and expertise among the studies. Surgical outcomes in DIEP flap reconstruction can be highly dependent on the surgeon’s skill and experience, factors that are difficult to quantify and control for in a narrative review. The review also predominantly focuses on short-term outcomes, such as immediate postoperative complications and hospital stay duration. This leaves a gap in understanding the long-term consequences of different drainage strategies, particularly regarding aesthetic outcomes, flap viability, and patient satisfaction. Furthermore, the patient populations in the reviewed studies may not fully represent the diversity of breast reconstruction patients. Factors such as differing BMI ranges, comorbidities, and demographic variations can significantly impact surgical outcomes and complications, yet these factors are not consistently accounted for in the existing literature [33]. Lastly, the method of dissection in the DIEP flap procedure, a potential factor influencing seroma formation and fluid accumulation, was not extensively covered in the reviewed studies. The impact of different dissection techniques, such as electrocautery versus scalpel dissection, on postoperative outcomes remains an underexplored area in the current body of literature. The method of dissection in the DIEP flap procedure was not discussed in detail in the aforementioned literature. It may, however, be a confounding factor affecting seroma formation and thus drain impact. Electrocautery, which is used conventionally, is associated with less blood loss than knife dissection but may impact fluid accumulation afterward [37].

### 3.7. Future Research

Despite the advancements in DIEP flap breast reconstruction and the evolving understanding of CSD use, there remain significant gaps in our knowledge that future research must address to optimize patient outcomes. Prospective RCTs comparing the efficacy and safety of CSD against modern, drain-free techniques like progressive tension sutures are critically needed. These studies should stratify patients based on relevant variables such as age, BMI, comorbidities, and extent of surgery to determine if certain subgroups benefit more from specific techniques. Additionally, a focus on patient-reported outcomes, including pain, mobility, and overall satisfaction, is essential to comprehensively evaluate the impact of drainage strategies. Moreover, there is a need to investigate the optimal timing for drain removal in DIEP flap reconstruction. Research should aim to establish standardized, evidence-based criteria for drain removal, potentially reducing the variability in current practices. This could include exploring the correlation between drainage volume and the risk of complications like seroma and hematoma formation. The potential role of novel technologies and materials in reducing seroma formation and other complications should also be explored. For instance, the use of biocompatible adhesives or sealants as alternatives to traditional suturing methods could present innovative approaches to wound closure in DIEP flap reconstruction.

## 4. Conclusions

While closed suction drainage remains a prevalent practice for minimizing complications such as seroma formation, its use is juxtaposed against emerging drain-free techniques, such as progressive tension sutures, that show promise in reducing hospital stays and enhancing patient comfort. The review underscores the lack of consensus and standardized protocols in drainage management, reflecting the need for more rigorous, randomized controlled trials to establish evidence-based practices. Additionally, the exploration of patient-centered outcomes and the long-term impact of drainage strategies on aesthetic and functional results is crucial. This review not only illuminates the current state of knowledge in the field but also identifies critical gaps in the literature, guiding future research towards optimizing patient outcomes in DIEP flap breast reconstruction.

## Data Availability

The original contributions presented in the study are included in the article/Supplementary Materials; further inquiries can be directed to the corresponding author.

## Supplementary Materials

The following supporting information can be downloaded at: https://www.mdpi.com/article/10.3390/jcm13216586/s1, File S1: Narrative review checklist.
