# Supplementary figures and images for: Optimal Use of Drain Tubes for DIEP Flap Breast Reconstruction: Comprehensive Review
Source: J Clin Med. 2024 Nov 1;13(21):6586. doi: 10.3390/jcm13216586 (PMC11547150; doi:10.3390/jcm13216586)

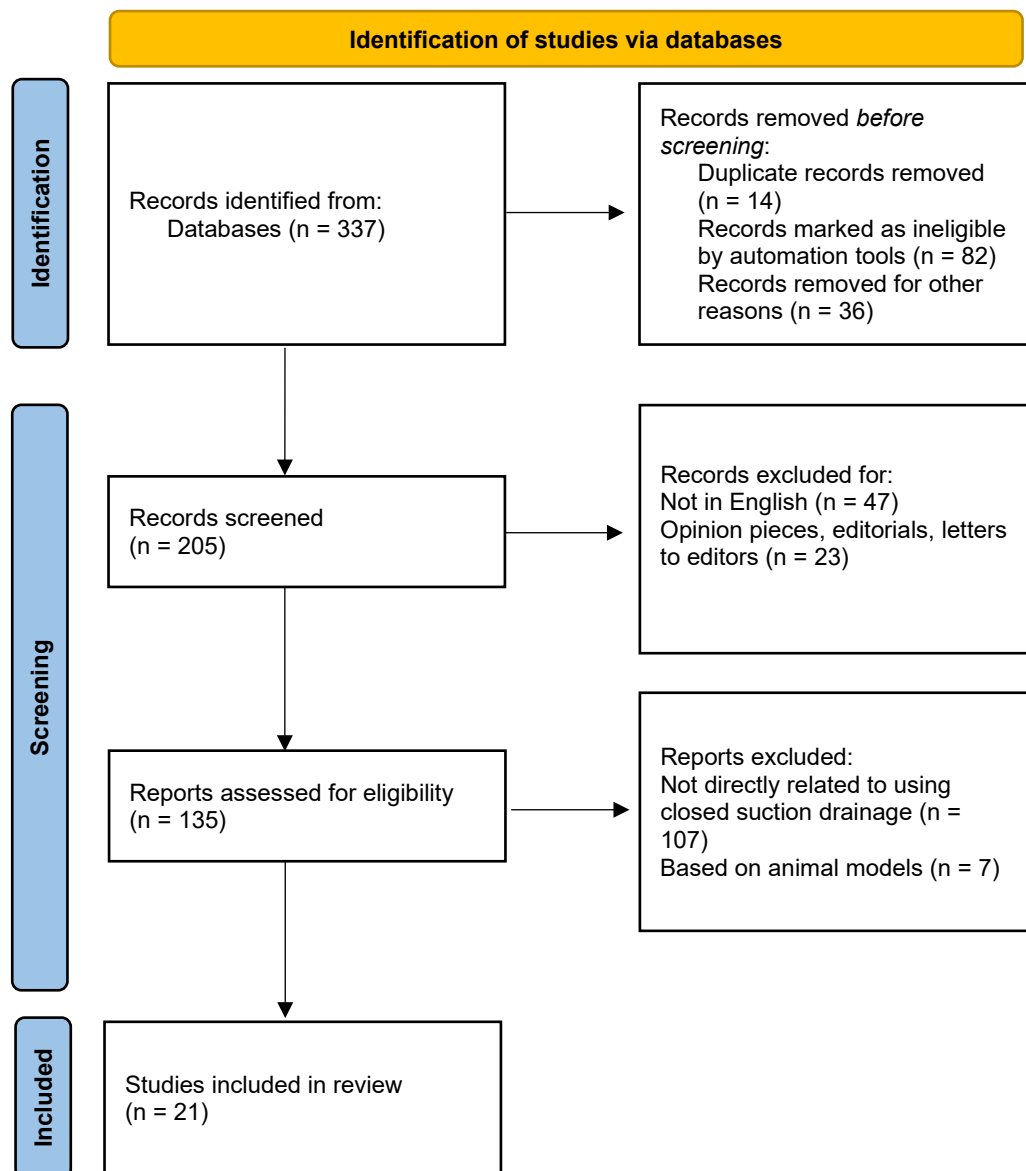

Supplement: Supplementary file 1 [file jcm-13-06586-s001.zip › jcm-3260758-supplementary.pdf]
